# Seed Treatment With Systemic Fungicides: Time for Review

**DOI:** 10.3389/fpls.2021.654512

**Published:** 2021-08-02

**Authors:** Mulla S. Ayesha, Trichur S. Suryanarayanan, Karaba N. Nataraja, Siddegowda Rajendra Prasad, Ramanan Uma Shaanker

**Affiliations:** ^1^Department of Crop Physiology, University of Agricultural Sciences, Bangalore, India; ^2^Vivekananda Institute of Tropical Mycology, Ramakrishna Mission Vidyapith, Chennai, India; ^3^Department of Seed Science and Technology, University of Agricultural Sciences, Bangalore, India; ^4^School of Ecology and Conservation, University of Agricultural Sciences, Bangalore, India

**Keywords:** fungal endophyte, seedling growth, carbendazim, bavistin, seed microbiome, holobiome, pathogen, seed endophytes

## Abstract

Pre-sowing seed treatment with systemic fungicides is a firmly entrenched practice for most agricultural crops worldwide. The treatment is intended to protect the crop against seed- and soil-borne diseases. In recent years, there is increasing evidence that fungicidal applications to manage diseases might inadvertently also affect non-target organisms, such as endophytes. Endophytes are ubiquitously present in plants and contribute to plant growth and development besides offering resistance to biotic and abiotic stresses. In seeds, endophytes may play a role in seed development, seed germination, seedling establishment and crop performance. In this paper, we review the recent literature on non-target effects of fungicidal applications on endophytic fungal community and discuss the possible consequences of indiscriminate seed treatment with systemic fungicide on seed endophytes. It is now well recognized that endophytes are ubiquitously present in all parts of the plant, including the seeds. They may be transmitted vertically from seed to seed as in many grasses and/or acquired horizontally from the soil and the environment. Though the origins and evolution of these organisms in plants are a matter of conjecture, numerous studies have shown that they symbiotically aid in plant growth and development, in nutrient acquisition as well in protecting the plants from abiotic and biotic stresses. Against this background, it is reasonable to assume that the use of systemic fungicides in seed treatment may not only affect the seed endophytes but also their attendant benefits to seedling growth and establishment. While there is evidence to indicate that fungicidal applications to manage plant diseases also affect foliar endophytes, there are only few studies that have documented the effect of seed treatment on seed-borne endophytes. Some of the convincing examples of the latter come from studies on the effect of fungicide application on rye grass seed endophyte AR37. More recently, experiments have shown that removal of seed endophytes by treatment with systemic fungicides leads to significant loss of seedling vigour and that such losses could be partially restored by enriching the seedlings with the lost endophytes. Put together, these studies reinforce the importance of seed endophytes to seedling growth and establishment and draw attention on how to trade the balance between the benefits of seed treatments and the direct and indirect costs incurred due to loss of endophytes. Among several approaches, use of reduced-risk fungicides and identifying fungicide-resistant endophytes are suggested to sustain the endophyte contribution to early seedling growth.

## Introduction

Seed treatment with systemic fungicides is a routine integrated crop management practice for crops. Despite the benefits realized by fungicide seed treatment including improved seed emergence, plant vigour and protection from seed- and soil-borne fungal pathogens (Lamichhane et al., 2020), recent studies have raised some concerns regarding this practice (Vasanthakumari et al., 2019; You et al., 2020). Of particular concern is the off-target effects of such treatment on the seed-borne microbiome, especially on fungi (Karlsson et al., 2014; Prior et al., 2017). Both culture-dependent and independent methods have demonstrated that there is a rich diversity of fungi inside seeds (Kinge et al., 2019; Chun et al., 2021). These fungal endophytes (FE) may have a role in early seedling growth and establishment. Considering that the seed microbiota serves as an important link between the maternal sporophyte generation and the next seedling generation, it is important to assess the consequences of seed treatments on these processes.

This paper offers a critical review of the practice of seed treatment with systemic fungicides. First, we trace the history of seed treatment and discuss briefly the different types of fungicides and their modes of action. Second, we highlight the possible role of seed microbiome in basic physiological processes, such as seed germination, seedling growth and establishment, and how such effects might be affected by systemic fungicides. We draw upon the literature that have examined the effects of fungicidal treatments on plants and seeds as well as their effects on non-target organism, including endophytes. Finally, we discuss the need to reduce indiscriminate use of seed treatment with systemic fungicides, which have adverse consequences on seed endophytes and seed health.

Disinfection of seed can be traced to as early as the 17th century, when wheat seeds were treated with brine solution to free them of smut caused by *Ustilago* (Tillet, 1755). In 1807, Prevost showed that dilute copper sulphate solution reduced seed-borne smuts and this practice became the main treatment throughout the 19th century. Following the establishment of the International Seed Testing Association in the late 1920s and the increased awareness of the damage caused by seed-borne pathogens, both during storage and post-seedling development, new seed treatment options were developed. The first contact fungicide, Captan, was used in seed treatments in the 1950s to protect seeds against a variety of fungal pathogens (Kittleson, 1952). This class of fungicide inhibited fungi from entering the plant tissue. At about the same time, the efficacy of methylmercury for the treatment of small grains was also recognized. However, due to environmental concerns, its use was discontinued in the early 1970s (Birah et al., 2014). The discovery in the early 1970s of systemic fungicides, such as carboxin and thiabendazole, which not only reduced seed-borne pathogens but also soil-borne pathogens, made them the choice for seed treatment. Systemic fungicide treatment of seed is an important strategy in disease management for many field and vegetable crops worldwide (Bhushan et al., 2013; Lamichhane et al., 2020).

One of the most commonly used systemic fungicides to manage fungal diseases is carbendazim, a methyl benzimidazole carbamate (MBC) group of fungicides. It was introduced and registered under USEPA in 1973 (Campos et al., 2015). The MBCs, which include bavistin and benomyl, bind to β-tubulin in microtubules and interfere with spindle fibre proliferation, resulting in the suppression of cell division. MBC is used in pre- and post-harvest applications to protect a wide class of both agricultural and horticultural crops, such as beet, banana, cereals, fodder rapeseed, mango, oranges, pomes, pineapples, strawberries, medicinal herbs, turf grasses and ornamental plants (Tortella et al., 2013; Singh et al., 2016). It is also used in combination with several other fungicides, such as mancozab, to manage fungal disease in mango and sunflower (Devi et al., 2015; Singh et al., 2016). Several other classes of fungicides, such as triazoles, phenylpyrroles, phenylamides, benzimidazoles and strobilurines, are also used for seed treatment (Zeun et al., 2013).

In many countries including the United States, Australia and France, pre-sowing fungicidal treatment of field crops is a routine practice (White and Hoppin, 2004; Agreste, 2019; Lamichhane et al., 2020; You et al., 2020). However, in these countries, there has been an increasing emphasis on the use of reduced-risk fungicides which have a high specificity for target organisms (Adaskaveg et al., 2005; Udayashankar et al., 2012). In India, the annual consumption of MBC fungicide is more than 2,000 metric tons (Singh et al., 2016) and it is registered for use in 18 crops including apple, bean, brinjal, barley, mango, cucurbit, cotton, grape, groundnut, jute, pea, paddy, rose, sugar beet, wheat, walnut and tapioca (Bhushan et al., 2013).

Seed treatments are generally provided before sowing as seed dressing, seed coating or seed pelleting (Pedrini et al., 2017). In seed dressing, which is the most common method of seed treatment, the seeds are dressed either with dry or wet formulations of fungicides and pesticides. Additionally, seeds are treated with natural bio-formulants like *Pseudomonas, Trichoderma* and *Rhizobia* to enhance their field performance. Seed coating is usually undertaken by industries for large lots of seeds and seed pelleting is practiced for crops with small seeds, such as carrots and onions (Tamil Nadu Agritech portal, 2020).^1^ Seeds may also be treated at the time of harvest to maintain quality during seed storage and transport.

## Fungal Endophytes of Seeds

The successful association between two eukaryotes belonging to two different Kingdoms (the fungal endophytes (FE) and their plant hosts) is not inconsequential (Krings et al., 2009). FE residing in tissues of a plant can enhance the plant’s ecological fitness by increasing its tolerance to pests (Raman and Suryanarayanan, 2017) and pathogens (Busby et al., 2016), and abiotic stresses like salinity (Sampangi-Ramaiah et al., 2020), high temperature (Ali et al., 2018; Sangamesh et al., 2018) and drought (Rodriguez et al., 2008). Although a sensu stricto definition of FE based on their taxonomy and mode of dispersal identifies different types (Rodriguez et al., 2009),here wefollow a broader definition as fungi inhabiting seeds internally without causing apparent harm to the seed or crop.

Postulated to have evolved from a pathogenic ancestry, FE produces no disease symptoms and occurs in the apoplastic spaces of the seed tissues. Although a plant can harbour FE in all its tissues, the species composition of the FE assemblage differs among the different tissues of individual plants (Suryanarayanan and Vijaykrishna, 2001). This is true for the seed FE as well (Geisen et al., 2017). Seed FEs are located in the seed coat, integument and rarely in the endosperm and cotyledon or the embryo (Philipson and Christey, 1986). Their mobilization into the seed tissues could occur vertically from parent to seeds, as in some cool-season grasses (Afkhami and Rudgers, 2008), in which case the endophytes move into the ovule and embryo through the caryopsis. Alternatively, FE may be transmitted horizontally; in such cases, endophytes gain entry into the phyllosphere through stomatal opening or physical injuries and then spread to various parts of the plant (Barret et al., 2016). Since the sieve tubes in the maternal tissues (seed coat and integument) and the offspring tissue (endosperm and embryo) of seed are not connected (Thorne, 1985), endophyte is generally not present in the latter tissue. Fungi from soil also infect fallen seeds and are retained and spread to the aerial tissues as endophytes (U’Ren et al., 2009).

Both culture-dependent and independent (metagenomic) analyses have revealed a rich diversity of bacterial and fungal endophytes in seed tissues (Shahzad et al., 2018; Kinge et al., 2019; Chun et al., 2021). It is believed that the seed microbiome (endophytes) is the first to be mobilized into a growing seedling, before it receives endophytes from the soil litter or through wind distribution (Mitter et al., 2017). Using metagenomics analysis, Chen et al. (2020) demonstrated that a greater diversity and density of seed-vectored microbes in rice may benefit seedlings by helping them tolerate stress and counter disease-causing organisms. Delinting of cotton seeds by acid treatment is done to facilitate easier mechanical planting. This process removes the cotton fibre-borne microbes, leading to increased susceptibility of seedlings to pests and pathogens (Irizarry and White, 2017). Long-term cultivation involving seed cleaning of wild tobacco (*Nicotiana attenuata*) eliminated the associated microbes, making the seedlings susceptible for fungal pathogens (Santhanam et al., 2015). Functional annotation of genes of endophytes associated with finger millet indicates their involvement in many plant growth and development responses, including abiotic and biotic stress tolerance, secondary metabolism, aromatic compound synthesis, and the glutathione and cysteine synthesis pathways (Prasannakumar et al., 2020). In fact, considering the overarching role of fungal endophytes in plant growth and development, it is clear that they play an important role in sustainable agriculture (Lugtenberg et al., 2016).

## Effects of Fungicide Treatment on Fungal Endophytes

In light of the increasing evidence of the role of endophytes on plant growth and stress tolerance, their use in real world agriculture could be constrained by the practice of seed treatment with fungicide (Murphy et al., 2017). Although seed treatment could include application of fungicides, insecticides or rodenticides, the majority of seed treatments is with fungicides (White and Hoppin, 2004). Seed treatments are *sine qua non* for managing diseases to increase stand establishment, seed yield and quality (Rothrock et al., 2012). Indeed, the practice of treating seeds with fungicides has increased many fold over the years (Urrea et al., 2013). While the major aim of seed treatments with fungicides is to bring down the pathogen load on the seed surface or inside without affecting seed viability and seedling fitness, several studies have cast doubts if this is indeed the case. Since environmental filtering and maternal factors determine the constitution of the fungal microbiome in seed (Fort et al., 2019), the effects of seed treatment with systemic fungicides on the seed endobiome and their consequences on seed and seedling performance need to be addressed.

In recent years, there is mounting evidence to suggest that foliar application of fungicides significantly affects non-target organisms, such as the endophytic fungi (Table 1). Fungicide application on wheat plants leads to significant differences in relative abundance and diversity of non-target fungi (Karlsson et al., 2014) and also inhibited the growth of endophytic yeast and filamentous fungi (Wachowska et al., 2013). For example, fungicide treatment affects the diversity of epiphytic and endophytic fungi in *Phaseolus vulgaris* (Prior et al., 2017). Comparing soybean grown using conventional plant protection versus those cultivated organically, Da Costa Stuart et al. (2018) reported a one-third reduction in foliar endophytes in the former. Batzer and Mueller (2020) reported differential effects of fluxapyroxad and pyraclostrobin sprays on *Diaporthe* and *Alternaria* endophytes; the fungicides significantly increased the proportion of endophyte species belonging to *Diaporthe* but decreased those of *Alternaria.* Besides affecting endophytic fungi, application of foliar fungicides and other plant protectants also reduce the endophytic proteobacteria (Chen et al., 2020).

**Table 1:** Studies highlighting the role of fungicide application on endophytes.

S. No.	Plant	Application	Effect on endophytes	Reference
1	Tall Fescue	Foliar spray	Significant reduction in leaf endophytic load	Williams et al., 1984
2	Tall Fescue	Foliar spray	Seedling endophyte abundance rates were higher when terrazole or chloroneb was applied compared with no fungicide or propiconazole	Hill and Brown, 2000
3	Rye grass and tall Fescue	Seed treatment	Endophyte load was reduced by more than 60% in leaf sheath	Leyronas et al., 2006
4	*Mangifera indica*	Foliar spray	Reduction in the colonization frequency of fungal endophytes in leaves	Mohandoss and Suryanarayanan, 2009
5	Rye grass	Foliar spray	*Neotyphodium* endophyte AR37 transmission into germinating seedlings was reduced by two different de methylation-inhibitor fungicides	Chynoweth et al., 2012
6	Wheat	Foliar application	Inhibited the growth of endophytic yeast-like and filamentous fungi on wheat kernels	Wachowska et al., 2013
7	Wheat	Foliar application	Causes significant difference in the relative abundance and diversity of non-target fungi in wheat leaves	Karlsson et al., 2014
8	Barley	Seed dressing	No effect on seed endophytes offered as seed dressing; improved seedling growth	Murphy et al., 2017
9	Oats	Seed dressing	No effect on seed endophytes offered as seed dressing; improved seedling growth	Murphy et al., 2017
10	*Phaseolus vulgaris*	Foliar application	Changes in the composition of epiphytic and endophytic community in leaves	Prior et al., 2017
11	*Vicia faba*	Foliar application	Changes in the composition of epiphytic and endophytic community in leaves	Prior et al., 2017
12	Perennial ryegrass	Foliar spray	No detrimental effect on AR37 endophyte content in seed	Cruz et al., 2018
13	Tomato	Root drenching	No effect on root endophyte	Malandrakis et al., 2018
14	Grapevine	Hot water dipping in combination with fungicide (stem cutting treatment)	Reduced incidence of endophytic fungi in the stem cuttings	Gorur and Akgul, 2019
15	Grapevine	Foliar spray	Wood mycobiome of grapevine cuttings is significantly affected by fungicide application	Del Frari et al., 2019
16	Rice	Soil application	No detrimental effect on root endophytes	Shen et al., 2019
17	Soybean	Foliar application during pod setting	Affects endophytes differentially; *Alternaria* increased while *Diaporthe* spp. decreased in leaves and stems	Batzer and Mueller, 2020
18	*Nicotiana tabacum*	Seed dressing	Reduces prevalence of seed bacterial endophytes	Chen et al., 2020
19	Wheat	Media amended with fungicides	Two dark septate endophytes, namely, *Alternaria alternata* and *Cochliobolus* sp. were tolerant to glyphosate, carbendazim and cypermethrin *in vitro*	Spagnoletti and Chiocchio, 2020
20	Tea plant	Foliar spray	Reduction in colonization of treated tissues (bark, xylem, old leaves and new leaves)	Win et al., 2021

Though less documented, seed treatment with fungicides could lead to similar loss or disruption of the seed microbiome including the endophytes compromising seed germination and early seedling development (Lugtenberg et al., 2016). For example, in rye grass and tall fescue, seed treatment with fungicides reduced endophyte loads by over 60% (Leyronas et al., 2006). Seedling endophyte abundance in rye grass was always higher when no fungicides were applied (Hill and Brown, 2000). Fungicide application reduced the vertical transmission of *Neotyphodium* endophyte AR37 into germinated seedlings of rye grass (Chynoweth et al., 2012), although Cruz et al. (2018) reported no detrimental effect of fungicide application on AR37 endophyte content in rye grass seeds. Thus, whether through foliar application, as most studies have demonstrated, or through seed treatments, there is evidence to suggest that fungicides adversely affect the endophyte load and composition of plants and seeds, possibly impairing the ecological fitness of plants (Nettles et al., 2016).

Since endophytes are inextricably embedded in the plant tissue, unravelling their role in seedling growth is problematic. Nevertheless, several studies have attempted to cleanse the seed using systemic fungicides to examine the effects thereof on seedling growth attributes. Vasanthakumari et al. (2019) examined the effect of pre-sowing fungicidal treatment on seedling growth of rice, green gram, soybean and cowpea. In all these crops, treatment with 0.2% bavistin eliminated the endophytes and reduced seedling growth and vigour compared to untreated seed in the absence of disease. The reduced seedling growth in rice was partially restored upon enrichment of the seedlings by a consortium of endophytes obtained from untreated seeds. These results strongly suggest that the decrease in seedling growth upon fungicide treatment is due to the loss-of-function associated with the endophytes, rather than to the phytotoxicity of the fungicide. In another study, Puente et al. (2009) found that seedling establishment was impaired in cactus seeds disinfected with antibiotics. Inoculation of antibiotic-treated cactus seedlings with bacteria isolated from control seeds restored seedling vigour, as reflected by the increased number of root hairs and average root numbers per seedling. Similar results were reported by Verma et al. (2017, 2018) for bacterial endophytes of rice seeds. Re-inoculation of endophytic bacteria isolated from control seeds resulted in partial recovery of seedling growth. Since MBC fungicide effects are not fungal species-specific, it is likely that the fungicide treated seeds when sowed may eliminate certain keystone soil fungal species as well leading to cascading effects on the ecosystem (Zotti et al., 2020). It is pertinent to note that limited information is available on the dynamics of migration of seed endophytes to the soil and *vice versa,* and this could determine seed endobiome composition (Nelson, 2018).

The results of a few studies allow us to speculate on the negative effects of treating seeds with systemic fungicides. The significant reduction of germination in bavistin- and thiram-treated wheat seeds was due of their inability to mobilize stored starch in the absence of endophytes (Gogna et al., 2015). The increased propensity of citrus, banana and leather leaf fern for infection by virulent pathogenic strains after application of benolate could be because of the lowered defences in the absence of endophytes (Kloepper et al., 2013). Though direct evidence is lacking, the results obtained for leaf tissues in this regard bolster such a hypothesis. Mango leaves treated with hexaconazole, a broad spectrum triazole systemic fungicide, became infected by FE species that could not infect untreated leaves (Mohandoss and Suryanarayanan, 2009). Another recent study shows that fungicide treatment alters the density of the native endophyte communities as well (Batzer and Mueller, 2020). Considering the ability of FE to produce antifungal and antibacterial compounds as well as phyto hormones (Santos et al., 2015; Hamayun et al., 2017; Bian et al., 2021), it is conceivable that a fungicide-induced disturbance in the community of native FE in the leaf affects host plant traits (Suryanarayanan, 2020).

Since fungicides have direct effects on plant metabolism, all fungicide-induced effects cannot be attributed to the elimination of endophytes by the chemicals. It is known that high concentrations of fungicides can disrupt plant metabolism. Storing seeds after treatment with fungicide for long periods can result in phytotoxicity (Lamichhane et al., 2020). Similarly, at higher concentrations, benomyl inhibits root mitotic activity (Dane and Dalgic, 2005). Many fungicides reduce root nodule development (Martensson, 1992) and reduce the development of mycorrhizal fungi (Menge, 1982). Fungicide application also reduces carboxylation efficiency and regeneration of ribulose1,5 bisphosphates and thus affecting CO_2_ assimilation (Dias, 2012).

## Research Gaps

**Figure 1:**
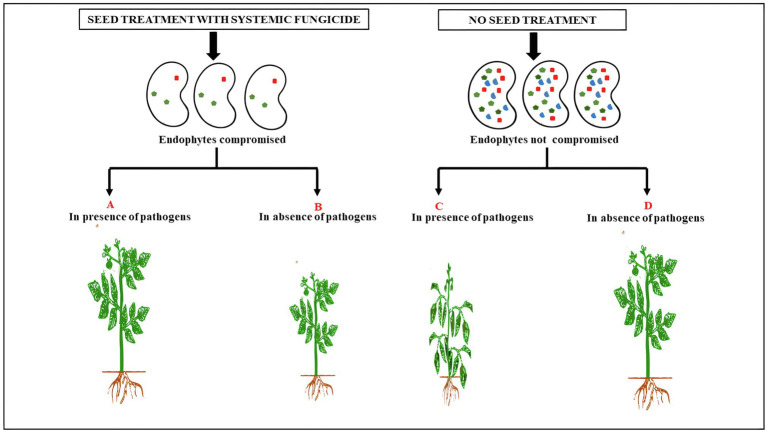
The effect of seed treatment with systemic fungicide on plant growth in presence or absence of seed- and soil-borne pathogens. The decrease in plant growth **(B)** represents the loss of endophyte-mediated growth promotion in seeds treated with systemic fungicide in the absence of seed- and soil-borne pathogens. This has to be contrasted with the phenotype of plant in **(D)**. The difference in growth between (B) and (D) can be attributed to the beneficial effects of endophytes which are lost due to seed treatment with systemic fungicides. (A,C) refers to plants subjected to soil and seed borne pathogens but either treated (A) or untreated (C) with fungicide.

Until recently, it was thought that seedlings acquire their symbiotic microbes from the soil, so the seed microbiome was studied, if at all, only for the presence of pathogens. It is now clear that seeds carry abundant fungi and bacteria as well as some Archaea as endophytes (Wassermann et al., 2019) and that a plant-specific core of microbiota is transmitted by seeds (Berg and Raaijmakers, 2018). With increasing evidence of the role of seed microbiome (especially endophytes) in seed development, seed germination and seedling growth, the merits of the century-old practice of systemic fungicidal seed treatment are now being questioned (Vasanthakumari et al., 2019). Have such treatments done more harm than good? What are the effects of fungicide combinations on non-target microbes and the ecosystem services they provide? Should seed treatment be a default option or based on anticipated risks? Are there alternatives to circumvent the effect of fungicides on seed endophytes? These and many other questions need some critical analysis (Figure 1). Just as the indiscriminate use of antibiotics has adverse consequences on the gut microflora (Antunes et al., 2011), routine fungicidal seed treatment in the absence of significant pathogen load could reduce crop performance and productivity. Simple risk assessment based on the growing conditions could potentially eliminate the use of millions of tonnes of fungicide and thereby help not only to sustain seed endophytes but also de-burden the environment of one source of chemical pollution (Lamichhane et al., 2020). Thus, it might be prudent not to view systemic fungicide seed treatments as routine and indispensable insurance against risks of crop failure, but rather as a choice depending on whether crop growth conditions are ideal or not (Alberta, 2021).^2^

The effect of fungicide seed treatment on the environment and non-target organisms is not well known. Although a few studies address the effect of fungicides on non-target soil microbes (Chen et al., 2001) and carbon and nitrogen cycling in soils, soil respiration, and nitrogen pools (Ullah and Dijkstra, 2019), there are hardly any investigations that quantitatively analyse the net benefits of fungicidal seed treatments. Gaspar et al. (2014) showed that fungicidal seed treatment of soybean did not significantly increase seed yield compared to untreated control. Thus, additional investments made in seed treatment might result in net economic loss. More meta-studies on different crops and regions are required to statistically validate the net economic returns of seed treatments.

The effect of long-term use of fungicides on endophytes of crops is not known (White et al., 2019). It is time that we reinvestigate well-known gains from fungicidal seed treatment, such as disease reduction (Russell, 2005), augmented field germination, seedling vigour (Babadoost and Islam, 2003), seedling stand and establishment (Loehken, 1990; Bradley, 2008), in the light of loss of endophyte microbes and environment quality.

Since seed microbiome influences plant protection as well as contributes to its ecosystem flexibility and diversification (Wassermann et al., 2019), knowledge on the role and metabolic function of endophytes in seeds could open up new possibilities for crop improvement. Studies in this direction are limited, partly because of methodological constraints in freeing seeds of their endophytes. Nonetheless, understanding the role of endophytes in early seed development, seed germination and seedling growth might allow for development of alternative options to seed treatment with fungicide. For instance, treating seedlings with foliar sprays of the endophytes critical to growth and development could be explored. Alternatively, identification of endophytes with tolerance to the applied fungicide and possessing the positive traits of the sensitive endophytes could allow seed treatments to be continued without impairment of the endophyte-induced functions (Murphy et al., 2017; Shen et al., 2019). The use of reduced-risk fungicide with narrower activity and targeted against specific pathogens could also alleviate the problems imposed by broad spectrum conventional fungicides (Adaskaveg et al., 2005; Udayashankar et al., 2012). The consequences of seed treatment with fungicides could also vary among plant species. For example, in the case of vertically transmitted endophytes, seed treatments could impose a greater penalty than in the horizontally transmitted endophytes. Walking the thin line between controlling the disease-causing organisms and conserving the beneficial organisms in the seeds opens up new challenges and calls for a greater understanding of these processes to reach a win-win situation.

## Conclusion

Management of seed- and soil-borne pathogenic fungi using fungicides is important for ensuring food security (Steinberg and Gurr, 2020). It is only recently that the crops have been recognized as a *holobiome* consisting of the plant and all its associated microbes. This has led to the suggestion of conserving seeds along with their associated microbes, such that these microbes are not lost forever due to the global practice of seed treatment (Berg and Raaijmakers, 2018). We reviewed the trade-off of pre-sowing seed treatment in defending seeds against seed- and soil-borne pathogens on the one hand and the possibility of losing seed benefiting endophytes on the other. Considering the potentially important role of seed-borne endophytes in seed germination and seedling growth, and being a source of endophyte inoculum for the different tissues of the developing plant, the century-old practice of routine seed treatment should be revisited. The gain accrued by seed treatment in disease management versus the potential loss in crop performance due to disturbance of seed endobiome by seed treatment should be studied for more crops using fungicides exhibiting different modes of action.

## Author Contributions

MA and RS planned the review. MA, RS, TS, KN, and SP drafted the manuscript. MA developed the figure. All authors contributed to the article and approved the submitted version.

## Conflict of Interest

The authors declare that the research was conducted in the absence of any commercial or financial relationships that could be construed as a potential conflict of interest.

## Publisher’s Note

All claims expressed in this article are solely those of the authors and do not necessarily represent those of their affiliated organizations, or those of the publisher, the editors and the reviewers. Any product that may be evaluated in this article, or claim that may be made by its manufacturer, is not guaranteed or endorsed by the publisher.

## Footnotes

^1^ http://agritech.tnau.ac.in/

^2^ https://www.alberta.ca/use-of-new-seed-treatments.aspx
